# Novel Application of 3D Scaffolds of Poly(E-Caprolactone)/Graphene as Osteoinductive Properties in Bone Defect

**DOI:** 10.1055/s-0042-1755550

**Published:** 2022-11-09

**Authors:** Hendrik Setia Budi, Silvia Anitasari, Yung-Kang Shen, Marut Tangwattanachuleeporn, Prawati Nuraini, Narendra Arya Setiabudi

**Affiliations:** 1 Department of Oral Biology, Dental Pharmacology, Faculty of Dental Medicine, Universitas Airlangga, Surabaya, Indonesia; 2 Department of Dental Material and Devices, Dentistry Program, Faculty of Medicine, Universitas Mulawarman, Samarinda, Indonesia; 3 Department Medical Microbiology, Medical Program, Faculty of Medicine, Universitas Mulawarman, Samarinda, Indonesia; 4 School of Dental Technology, College of Oral Medicine, Taipei Medical University, Taipei, Taiwan; 5 Faculty of Allied Health Sciences, Burapha University, Chon Buri, Thailand; 6 Research Unit for Sensor Innovation, Burapha University, Chon Buri, Thailand; 7 Department of Pediatric Dentistry, Faculty of Dental Medicine, Universitas Airlangga, Surabaya, Indonesia; 8 Medical Program, Faculty of Medicine, Universitas Airlangga, Surabaya, Indonesia

**Keywords:** PCL, graphene, scaffold, osteoinductive, tissue engineering

## Abstract

**Objective** Scaffolds provided a surface on which cells could attach, proliferate, and differentiate. Nowadays, bone tissue engineering offers hope for treating bone cancer. Poly(e-caprolactone) (PCL)/graphene have capability as an osteogenic and regenerative therapy. It could be used to produce bone tissue engineering scaffolds. The purpose of this study was to investigate the ability of PCL/graphene to enhance the osteoinductive mechanism.

**Materials and Methods** The PCL/graphene scaffold was developed utilizing a particulate-leaching process and cultured with osteoblast-like cells MG63 at 0.5, 1.5, and 2.5 wt% of graphene. We evaluated the porosity, pore size, migratory cells, and cell attachment of the scaffold.

**Statistical Analysis** Data was expressed as the mean ± standard error of the mean and statistical analyses were performed using one-way analysis of variance and Tukey's post hoc at a level of *p* -value < 0.05.

**Results** Porosity of scaffold with various percentage of graphene was nonsignificant ( *p* > 0.05). There were differences in the acceleration of cell migration following wound closure between groups at 24 hours ( *p* < 0.01) and 48 hours ( *p* < 0.00). Adding the graphene on the scaffolds enhanced migration of osteoblast cells culture and possibility to attach. Graphene on 2.5 wt% exhibited good characteristics over other concentrations.

**Conclusion** This finding suggests that PCL/graphene composites may have potential applications in bone tissue engineering.

## Introduction

Typically, bone reconstruction or regeneration requires the use of a biocompatibility scaffold with a porous structure. The scaffold should possess sufficient strength to support the injured bone in place. A scaffold is generally able to control the proliferation of cells that have migrated from surrounding tissue or been seeded inside the porous surface of the scaffold. Therefore, the scaffold's pore and pore interconnectivity contribute to cell adhesion and proliferation, as well as to the transport of nutrients and oxygen throughout the three-dimensional (3D) constructs. 1 2

Previous studies have reported that scaffolds for bone repair must be biodegradable, biocompatible, porous, and have strong interconnectivity between pores for the cells to adhesion, proliferation, and differentiation. 3 4 Therefore, physical properties like porosity and pore size are important characteristic for the good scaffolds. On the one hand, a high porosity enhances water and nutrient absorption while decreasing mechanical characteristics. Hence, a suitable scaffold's porosity should be comparable to that of bone, such as cancellous bone (79.3). On the other hand, the pore size should regulate the growth of cells that have migrated from the surrounding tissue, which is suitable for cell growth and supporting cell activities such as nutrient uptake and waste processing. 5 6 7

Poly (e – caprolactone) (PCL) is one of the materials meeting these characteristics. In recent years, PCL has been utilized as a biomedical scaffold due to its biodegradable and biocompatible properties. However, this material's poor mechanical qualities and pore size, limit its use in bone engineering. 2 6 Therefore, it needs filler that can improve the scaffold's properties. 8 9

Graphene, a new allotrope of carbon, is characterized as a two-dimensional honeycomb lattice composed of carbon atoms in monolayers. Graphene and its derivatives have garnered considerable attention in the field of materials research since graphene has no visible toxicity and exhibits high biocompatibility. Indeed, *in vitro* experiments have demonstrated graphene's capacity to promote osteoblast proliferation and enhance their differentiation into mature osteoblasts. Thus, graphene is a promising material for enhancing the bioviability and bioactivity of synthetic scaffolds, especially in cooperation with polymer, such as PCL. 9 10 11

Not only are physical properties an important thing for a good scaffold, but the ability of cells to migrate is important too in a variety of physiological and pathological processes. *In vitro* cell migration can be studied using the wound healing test (scratch assay), which is a straightforward approach. 12 This method is based on the finding that when an artificial gap is made in a confluent cell monolayer, the cells on the edge of the gap begin to migrate until new cell-cell connections are formed. 13 14 The migratory and differentiation processes of cells are crucial for tissue engineering and regenerative medicine. Recently, the majority of efforts have been directed toward controlling cell destiny via manipulation of biophysical or pharmacological inputs. 15 Chemical cues like ligands, extracellular matrix (ECM) proteins, and biomolecules have all been shown to influence cell activity on chemically changed surfaces. 16

The purpose of this study was to disclose the physicochemical behavior of PCL and graphene at varied concentrations (0.5, 1.5, and 2.5 wt%) on osteoblast-like cells MG-63 in determining which concentrations to enhance the osteoinductive mechanism.

## Materials and Methods

### Fabrication of PCL/Graphene Scaffold

**Fig. 1:**
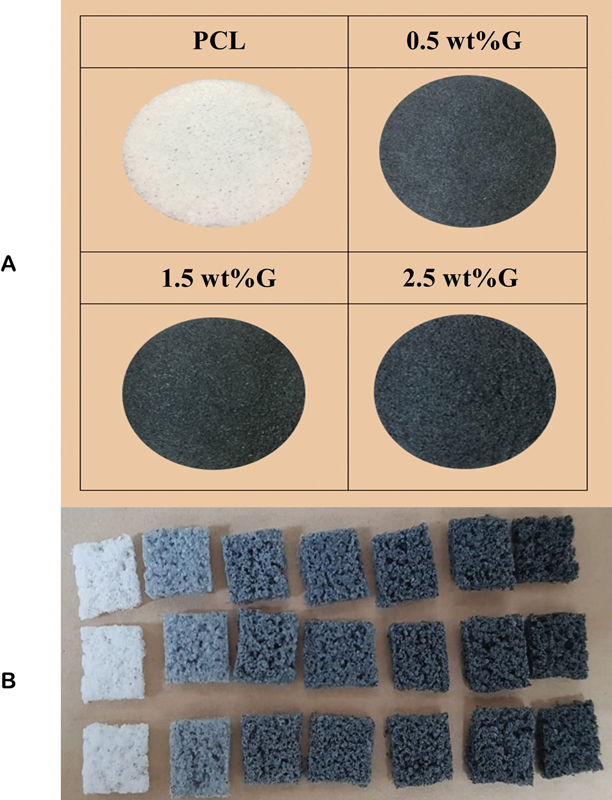
Scaffold fabrication. Group of poly(e-caprolactone) (PCL) without graphene (PCL); Group of PCL containing graphene at concentrations of 0.5 wt% (0.5 wt%G); Group of PCL containing graphene at concentrations 1.5 wt% (1.5 wt%G); Group of PCL containing graphene at concentrations of 2.5 wt% (2.5 wt%G). ( **A** ) PCL/graphene scaffold 10 × 10 × 2 mm ^3^ in size. ( **B** ) PCL/graphene scaffold 1 × 1 × 2 mm ^3^ in size.

3D porous scaffolds were manufactured in this study employing a solvent casting/particulate leaching process. The PCL (M _n_ 80,000) from Sigma-Aldrich (St. Louis, Missouri, United States) was used as the matrix material and natrium chloride (NaCl) as the porogen (Sigma-Aldrich). Graphene was produced by heating a graphite intercalation compound to 700°C in a common furnace, positioned in front of a fume closet to avoid inhalation of the nanoparticles, and leaving it there for 60 seconds. These layers grew by ultrasonication process, resulting in graphene dispersion in the solvent. For 12 hours at room temperature, PCL was dissolved in chloroform in a 1:10 w/v ratio. The PCL solution was then added to the NaCl and graphene solutions and stirred for 2 hours with a magnetic stirrer. The blended solution was poured into a mold and allowed to dry at room temperature for 1 day. Chloroform residues were eliminated during a 24-hour period in a vacuum oven set to 37°C. The PCL/graphene scaffolds were immersed in deionized (DI) water for 24 hours to eliminate the porogen, with the DI water being replaced every 2 hours throughout this time period. The PCL/graphene scaffold was then dried in a vacuum oven set to 50°C for 12 hours. Finally, a porous PCL/graphene-blended scaffold was developed. 16 We synthesized porous scaffolds PCL containing graphene at concentrations of 0.5, 1.5, and 2.5 wt%. The PCL/graphene is manufactured in a 10 × 10 × 20 mm ^3^ format ( Fig. 1 ).

### Porosity

Dried scaffolds were immersed in absolute ethanol for 2 hours and weighed after excess ethanol on the surface was blotted. The porosity was calculated using equation 5 6 :



where *M*_1_ and *M*_2_ are the mass of scaffolds before and after soaking in absolute ethanol, respectively; *ρ* is the density of absolute ethanol, and *V* is the volume of the scaffolds.

### Pore Distribution

A scanning electron microscope (SEM; Hitachi SU3500) was used to observe samples of 3D porous scaffolds. Gold was sputtered onto samples using a sputter coater in a vacuum chamber and then observed. Then, using Image J software, the SEM picture was analyzed to determine the pore size. 5 6

### Scratch Wound Assay (Migration)

The osteoblast-like cells MG-63 were cultured in 12-well plates. Around 3 × 10 ^4^ cells were seeded into each well and allowed to reach 90% confluency. Using a 200-μL tip, the cell monolayers were scratched and rinsed with phosphate-buffered saline (PBS) to remove detached cells and other debris. Three representative images from each of the scratched areas were photographed to estimate the relative migration of cells. The migration cell (scratch assay) was analyzed using inverted microscope (IX73, Olympus, Japan), in 100× magnification, and has been processed using Gen 5.0 software. The distance between the two edges of the wound sites was detected at 24 and 48 hours and analyzed by Image J software. Wound closure was calculated using the equatioon 17 :



### Cell Culture and Morphology

Osteoblast-like cells MG-63 were seeded onto PCL/graphene scaffolds to examine cell adherence and growth characteristics at various graphene weight ratios. First, osteoblast-like cells MG-63 were grown in Dulbecco's Modified Eagle Medium (Gibco) with 10% fetal bovine serum (Sigma, 12106C) and 1% penicillin (Gibco, 15140122). Cells were cultured in T75 flasks (37°C with 5% CO _2)_ in a cell culture incubator, and the media was replenished every 2 to 3 days. Each sample used in the cell culture was 10 × 10 × 2 mm ^3^ in size. Sterilization of samples was accomplished by soaking them overnight in 95% ethanol and then washing them twice with PBS (Gibco) to eliminate residual ethanol. After that, samples were transferred to 24-well plates. Cells were detached using 0.25% trypsin-ethylenediaminetetraacetic acid (Gibco) and each sample was seeded with 0.5 mL of a cell suspension at a concentration of 10 ^4^ cells/mL in 24-well tissue culture plates. For 21 days, the 24-well plates were incubated in a cell culture incubator. Throughout this period, the medium was renewed every 2 to 3 days. On days 7 and 21, samples were withdrawn to observe the results of cell culture. A SEM was used to determine the cell morphology. 16

### Statistical Analysis

Data was expressed as the mean ± standard error of the mean and statistical analyses were performed using one-way analysis of variance and Tukey's post hoc test to determine the relevant data differences using SPSS version 21.0 software (SPSS, USA). Significant differences between groups were determined at a level of *p* -value < 0.05.

## Results

### Porosity and Pore Size of Scaffold

**Fig. 2:**
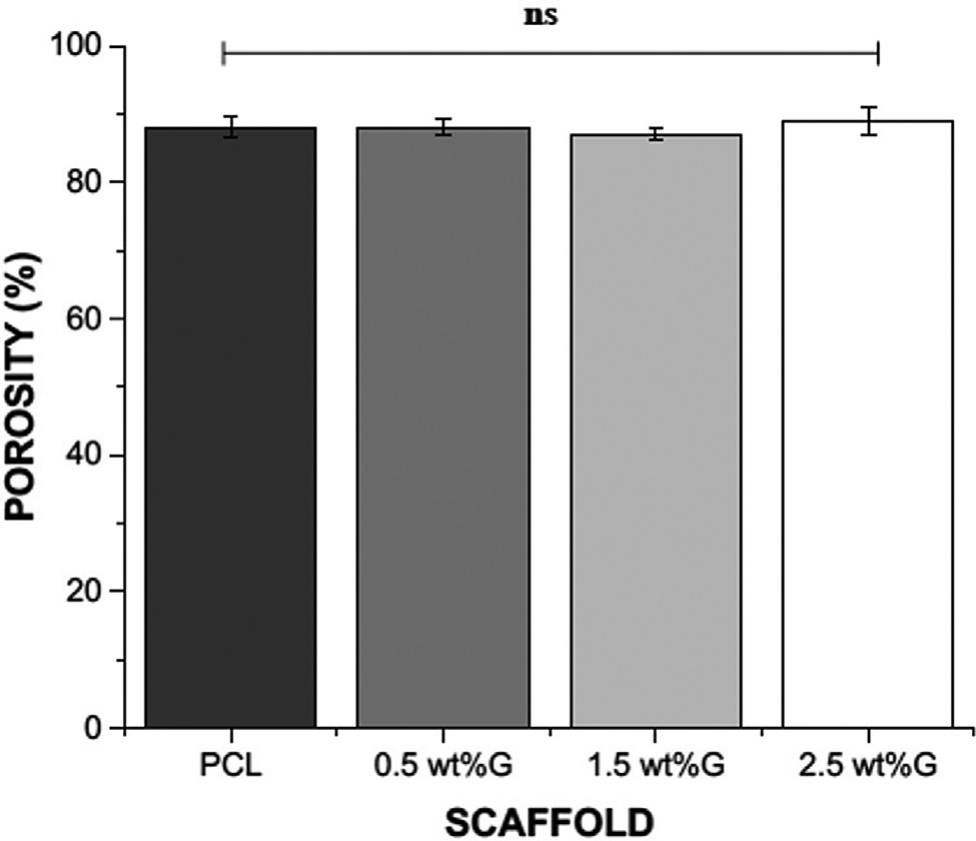
Porosity of scaffold with various percentage of graphene; ns, nonsignificance ( *p* > 0.05). Group of poly(e-caprolactone) (PCL) without graphene (PCL); Group of PCL containing graphene at concentrations of 0.5 wt% (0.5 wt%G); Group of PCL containing graphene at concentrations 1.5 wt% (1.5 wt%G); Group of PCL containing graphene at concentrations of 2.5 wt% (2.5 wt%G).

Physical properties, like as porosity and pore size are critical in determining the scaffold's quality. In this study, the porosity of PCL incorporated with various concentration of graphene had been similar with each other. PCL has porosity 88% (±1.5). The porosities of the PCL/graphene scaffolds with 0.5, 1.5, and 2.5 wt% were 88 (±1.2), 87 (±0.9), and 89 (±2.1) respectively ( Fig. 2 ). This finding was closed to porosity of cancellous bone (average 79.3%). 5 7

**Fig. 3:**
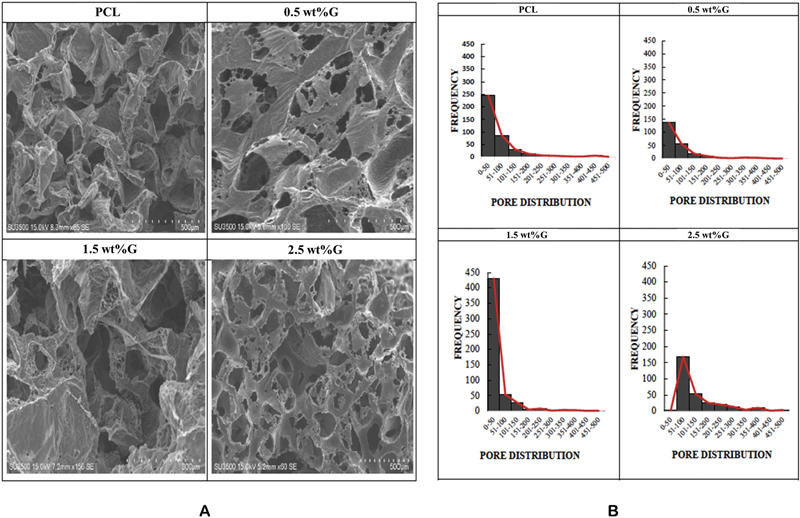
Morphology and pore distribution of poly(e-caprolactone) (PCL) and PCL/graphene analysis by scanning electron microscope (SEM). Group of PCL without graphene (PCL); Group of PCL containing graphene at concentrations of 0.5 wt% (0.5 wt%G); Group of PCL containing graphene at concentrations 1.5 wt% (1.5 wt%G); Group of PCL containing graphene at concentrations of 2.5 wt% (2.5 wt%G). ( **A** ) Pore distribution was determined by a scanning electron microscope. ( **B** ) Pore distribution of scaffold.

Pore distribution was determined by a SEM ( Fig. 3A ). It showed the porous size of these scaffolds ranged from 0 to 500 μm. The scaffold with a graphene concentration of 0.5 wt% has a pore size of 0 to 50 μm larger than others and PCL has a pore size of 400 to 450 μm larger than others, while the scaffold with a graphene concentration of 2.5 wt% has a pore size of 51 to 100, 101 to 150, 151 to 200, 201 to 250, 251 to 300, 301 to 350, 351 to 400, and 451 to 500 μm larger than PCL 0.5 and 1.5 wt% of graphene ( Fig. 3B ).

### Migration Enhancing of PCL/Graphene Composite to Osteoblast-Like Cell

To evaluate the osteoblast-like cells MG63 migration response, the cells were exposed to different graphene concentrations (0.5, 1.5, and 2.5 wt%) and allowed to migrate for 24 and 48 hours. Using a wound healing assay, we observed a graphene's concentration-dependent effect on osteoblast-like cells MG63 migration. The microscopic figure has to be analyzed to obtain information about the migration characteristics of the cultured cells. This could be accomplished manually by measuring the surface area and gap distance using image processing software such as Image J ( Table 1 ). The data show that the closest 0 µm gap between cells can be achieved on PCL/graphene 0.5 to 2.5 wt% composite at 48 hours. The farthest cell spacing was found using PCL/graphene 0.5 wt%.

**Table 1:** Cell migration at 0, 24, and 48 hours in the closure defect

Groups	The shortest distance (µm)			The longest distance (µm)			Closure defect (µm2)			*p* -Value a	
	0 h	24 h	48 h	0 h	24 h	48 h	0 h	24 h	48 h	24 h	48 h
PCL	165.0	53.9	24.1	323.9	323.9	237.7	668.1	587.7	402.4	0.01	0.00
PCL/Graphene 0.5		28.2	0		316.0	303.4		482.1	340.6		
PCL/Graphene 1.5		236.9	0		191.7	132.7		236.9	106.4		
PCL/Graphene 2.5		278.5	0		262.1	81.1		278.6	23.4		

Abbreviation: PCL, poly(e-caprolactone).

a There was a significant difference between groups in the area of closure defect on 24 hours ( *p* = 0.01) and 48 hours ( *p* = 0.00).

**Fig. 4:**
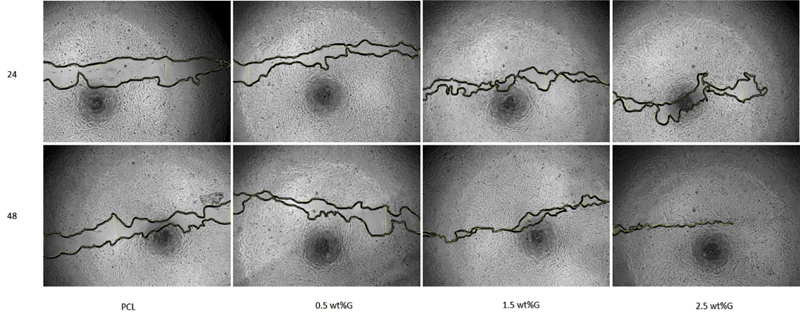
The effectiveness of the poly(e-caprolactone) (PCL)/graphene composite was measured by migration cell (scratch assay) using inverted microscope (IX73, Olympus, Japan), 100× magnification. Group of PCL without graphene (PCL); Group of PCL containing graphene at concentrations of 0.5 wt% (0.5 wt%G); Group of PCL containing graphene at concentrations 1.5 wt% (1.5 wt%G); Group of PCL containing graphene at concentrations of 2.5 wt% (2.5 wt%G).

The condition of 100% cell confluence was utilized to get the signal that the scratch defect had been completely closed. When treated with 2.5 wt% graphene, osteoblast-like cells MG63 showed nearly full closure of the scratch site within 48 hours, compared with the other treatments. As a result, a statistically significant increase in responsiveness was detected at 2.5 wt% graphene concentrations when compared with the others concentration and control ( Fig. 4 ).

### Cell Adherence on PCL/Graphene Scaffolds

**Fig. 5:**
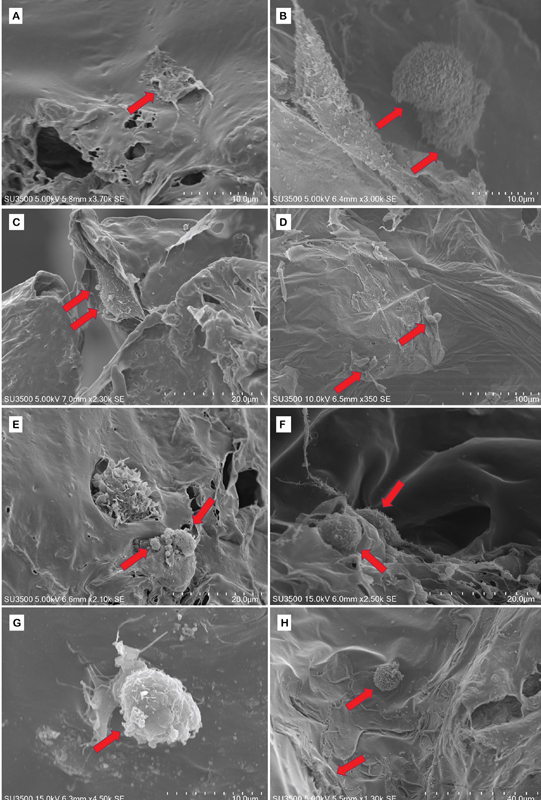
Osteoblast-like cells MG63 attachment to poly(e-caprolactone) (PCL)/graphene scaffolds were observed using scanning electron microscope (SEM) (Hitachi SU3500). ( **A** ) PCL 7 days, ( **B** ) PCL 21 days, ( **C** ) 0.5 wt%G 7 days, ( **D** ) 0.5 wt%G 21 days, ( **E** ) 1.5 wt%G 7 days, ( **F** ) 1.5 wt%G 21 days, ( **G** ) 2.5 wt%G 7 days, and ( **H** ) 2.5 wt% G 21 days. Red arrow is osteoblast-like cells.

Cells cultured on PCL and PCL/graphene scaffolds were obtained by SEM on days 7 and 14 ( Fig. 5 ). It demonstrated cells adhering to and multiplying within the pore, where each cell has a long filopodia that acts as a signaling pathway between the cells. On day 7, it was discovered that cells had adhered, proliferated, and early differentiated in cell culture. They were round in shape (in PCL with 1.5 and 2.5 wt% graphene), whereas PCL and 0.5 wt% graphene were flat and elongated. On day 21, differentiation revealed that the majority of cells on the scaffold had spherical forms. In other words, it showed how graphene changes more quickly when there is more graphene than when there is no graphene.

## Discussion

The total porosity of the scaffolds was measured in this study using the liquid displacement method. The constructions were more than 85% porous in total. When compared with cancellous bone (which have an average porosity of 79.3%), the porosity of the scaffolds is optimal since it promotes bone ingrowth without reducing mechanical qualities such as strength. 7 8

A variety of scaffolds made from different biomaterials and constructed utilizing a variety of fabrication techniques were used. Biocompatibility, biodegradability, mechanical qualities, scaffold architecture, and manufacturing technique are all key factors to consider when creating or establishing the appropriateness of a scaffold for use in tissue engineering. In a successful tissue engineering technique, these properties are deciding criteria when selecting a biomaterial. 18

Pore size, along with porosity, is an essential physical property for cell adhesion, gas diffusion, and the delivery of nutrients and wastes. In general, it has been noted that while the scaffold's large pore size or porosity promotes effective nutrition supply, gas transport, and metabolic waste disposal, it results in low cell attachment and intracellular signaling. While a small pore size or porosity may have the opposite effect, the optimal pore size is in the diameter range of 100 to 500 μm, which is thought to stimulate osteogenesis and angiogenesis due to the size of osteoblasts, which is approximately 10 to 50 μm. 9 12 19

Cell migration and proliferation are required for a range of physiological and pathological processes, including wound healing, 20 revascularization, cartilage regeneration, and bone regeneration. 21 22 Cell migration can be induced by biochemical and biophysical cues such as the mechanical properties of matrix, peptides, and growth factors in an immobilized and free form, respectively, which are referred to as mechanotaxis, haptotaxis, and chemotaxis. 23 Two types of biomaterials of PCL and graphene have been used to facilitate cell migration; one is scaffolds, which have a predetermined architecture, however cell infiltration is more difficult with scaffolds. 24 We assessed an enhancement of osteoblast migration by adding graphene starting on 24 hours, and closure of the scratch completely within 48 hours in comparison to the group of PCL. Adding graphene promoted osteoblast migration better than without graphene group, and in the future, it will have a greater impact on bone tissue regeneration applications. This is in accordance with a recent study by Du et al. 25

Because of their osteoinductive properties and antibacterial activity, graphene-based materials (GMs) have a bright future in bone tissue engineering. The GMs trigger osteogenic differentiation via a variety of mechanisms and routes. To begin with, mechanical stimulation from the porous folds of graphene or graphene oxide can launch a cascade of processes that enhance osteogenic development without the need of chemical inducers. In addition, GMs regulate osteogenesis through the ECM, macrophage polarization modulation, the oncostatin M signaling route, the mitogen–activated protein kinase signaling network, the bone morphogenetic protein signaling pathway, the Wnt/-catenin signaling pathway, and other pathways. 26

SEM images demonstrate cell attachment, proliferation, and differentiation. It demonstrated that osteoblast-like cells could adhere to, proliferate, and differentiate on the scaffold surface. 27 The proliferation of the cells was regulated by actin. It stimulates signal transduction and cell division. Myosin proteins interact with actin filaments to produce two distinct forms of movement. 28 29 To begin, myosin generates force between actin filaments, causing contractions that pull the rear of moving cells up, pinching cells in half, and reshaping them into tissues. 30 Muscle cells are contracted using a similar manner. Second, myosin associated with subcellular organelles and macromolecular protein and ribonucleic acid complexes transports these cargos over small distances along actin filaments. 31 32

The rapidly expanding science of tissue engineering for dental tissues will undoubtedly result in a dramatic shift in the availability of novel items for practitioners to employ on a daily basis. When implanted on the side of an injury, a scaffold material should strive for endogenous cell repopulation as well as recipient remodeling.

## Conclusion

PCL/graphene composites may have potential applications as novel bone tissue engineering. The porosity and pore size were suitable to induce osteogenesis and angiogenesis, and also stimulated osteoblast migration by adding graphene start from 24 hours.
